# The impact of Climate Adaptive Pilot Cities policy on urban pollution reduction and carbon emission mitigation

**DOI:** 10.3389/fpubh.2026.1803462

**Published:** 2026-04-10

**Authors:** Xin Wang, Mengyu Wang, Jiahan Hu, Hao Hu, Bingnan Guo

**Affiliations:** 1 School of Humanities and Social Sciences, Jiangsu University of Science and Technology, Zhenjiang, China; 2 College of Engineering, University of Perpetual Help System Laguna, Laguna, Philippines; 3 School of Economics, Shanghai University, Shanghai, China

**Keywords:** carbon emission mitigation, Climate Adaptive Pilot Cities, Green Technology Innovation, pollution reduction, Public Climate Risk Perception

## Abstract

Mitigation and adaptation are recognized as essential pillars for urban sustainability; however, the degree to which adaptation oriented policies contribute to broader environmental improvements remains an unresolved empirical question. This study treats the Climate Adaptive Pilot Cities (CAPC) initiative as a quasi natural experiment to evaluate its integrated impacts on urban pollution and carbon reduction. Utilizing a multi period staggered difference in differences framework on a comprehensive panel of 273 Chinese municipalities from 2011 to 2023, we find that the CAPC initiative significantly improves environmental outcomes, evidenced by a 0.284 decline in aggregate emission intensity. Mechanism analysis reveals that this reduction is driven by informal regulation via heightened public climate risk perception and innovation compensation via green technology advancement. Specifically, the policy initiates a state to society vertical diffusion of environmental norms, where pilot cities function as social amplification stations to foster a broad based social consensus. Notably, policy efficacy depends on local conditions: significant abatement effects are concentrated in high income cities with robust fiscal capacity, non-resource based cities free from carbon lock in, and central or western regions with vast untapped marginal abatement potential. These findings underscore the necessity of dedicated fiscal support and structural reforms to bridge urban resilience with low carbon transitions to ensure inclusive environmental governance.

## 1 Introduction

Climate-driven extreme events increasingly threaten human sustainability (1), requiring a synchronized advancement of mitigation and adaptation. Yet, urban governance often faces an inherent friction between pollution control and carbon mitigation, rooted in their distinct spatio-temporal scales and governing logic. While traditional pollution abatement (e.g., *SO*_2_) targets immediate localized health through territorial mobilization, carbon reduction serves global interests with long-term latency. Such mismatched priorities frequently trigger strategic conflicts and resource tradeoffs during policy implementation. Without a systemic framework, cities risk the “maladaptation” trap of defensive resilience efforts inadvertently exacerbating aggregate emission burdens (2–4). Therefore, bridging the barrier between adaptation and mitigation to generate Urban Pollution Reduction and Carbon Emission Mitigation (*UPCRL*) effects has become a global imperative (5).

To address these structural bottlenecks, the National Development and Reform Commission (NDRC) and the Ministry of Housing and Urban-Rural Development (MOHURD) jointly launched the Climate Adaptive Pilot Cities (CAPC) program in February 2017. By designating an initial batch of 28 pilot cities with diverse administrative levels and geographical regions, this initiative marks a strategic shift from “mitigation-led” governance toward “systemic adaptation” (6, 7). Unlike previous single-objective initiatives such as the Low-Carbon City Pilot (LCCP) or the Sponge City program (8, 9), the CAPC framework emphasizes integrating resilience into the full spectrum of urban planning, infrastructure investment, and industrial layout. While existing studies have extensively evaluated the effectiveness of CAPC in disaster resilience, supply chain stability, or green economic efficiency (10–13), there is little clarity on whether this adaptation-centric framework can drive concomitant reductions in pollution and carbon emissions. The potential for this policy to induce such ancillary environmental dividends remains an unexplored “black box” in current literature (2, 14).

Utilizing a comprehensive panel dataset of 273 Chinese municipalities over the 2011 to 2023 period, this study employs a multi-period staggered difference-in-differences (DID) framework to address this gap. Our contribution is three-fold: First, we quantify a substantial 28.4% decline in the integrated emission intensity of pilot cities, providing robust evidence for the cross-domain impacts of adaptation policies. Second, departing from a purely administrative perspective, we utilize natural language processing (NLP) to construct a Public Climate Risk Perception (PCRP) index based on government-citizen interaction data. This reveals the micro-logic of how the policy awakens public consciousness to form “informal regulatory” pressures, offering an innovation to the traditional Porter Hypothesis account (15). Finally, by identifying the boundary conditions of policy effectiveness, we provide evidence for constructing a regulatory system that harmonizes safety resilience with broader environmental performance improvements.

The remainder of this paper is structured as follows. Section 2 provides the detailed institutional background of the CAPC policy and develops the theoretical framework. Section 3 describes the data sources, the PCRP index construction, and the empirical strategy. Section 4 presents the baseline findings and a series of rigorous robustness checks. Section 5 explores the underlying “informal regulation” and “innovation compensation” mechanisms. Section 6 examines the heterogeneity of policy effectiveness across different urban archetypes, specifically addressing resource-based cities with high systemic inertia. Finally, Section 7 concludes and offers policy implication.

## 2 Policy background and research hypotheses

### 2.1 Policy background

The Climate Adaptive Pilot Cities (CAPC) program was formally initiated in February 2017 through a joint notice issued by the NDRC and MOHURD (NDRC Climate [2017] No. 343) (16). This initiative designated 28 pilot cities across diverse administrative levels and ecological zones to establish a representative framework for operationalizing resilience-building strategies. Unlike single-sector mandates, the CAPC framework integrates resilience targets into the full lifecycle of urban planning, infrastructure, and industrial realignment. In practice, the policy leverages monitoring and early-warning systems to enhance public awareness of climate risks (17), while utilizing adaptive standards and financial incentives to drive corporate technological upgrading. By prioritizing resilient infrastructure such as ecological buffer zones and low-carbon networks (18), the mandate seeks to mitigate systemic vulnerabilities. This institutional design provides a foundation for investigating whether such a resilience-centric model can overcome systemic inertia (19) and generate concomitant environmental dividends (20) beyond its primary safety objectives.

### 2.2 Research hypotheses

#### 2.2.1 Urban pollution reduction and carbon emission mitigation effect

We assume that the CAPC policy encourages pollution and carbon cut mainly due to a Resource Reallocation Effect. The policy causes the withdrawal of inefficient high-risk industrial capacities and direct factors of production such as capital and land into low-carbon resilient sectors through strict adaptive constraints imposed on urban planning. At the same time, the incentives which are flexible and entrench in the policy including green procurement and fiscal subsidy encourage the market entities to feed on clean production processes in order to produce to higher levels of adaptive standards. This package of limitations and incentives will then make a structural change and achieve the control of urban pollution reduction and carbon emission mitigation at the source (21). In light of the aforementioned logical deductions, the first hypothesis is formulated as follows.

**Hypothesis 1 (H1):** *The CAPC policy significantly promotes urban pollution reduction and carbon emission mitigation*.

#### 2.2.2 The informal regulation channel: costly signaling and social amplification of risk

Beyond the formal regulatory constraints described above, the CAPC policy exerts a profound influence through an informal regulation channel, specifically by reshaping Public Climate Risk Perception (*PCRP*). However, administrative slogans alone are often insufficient to overcome the public's cognitive inertia regarding long-term climate risks. We argue that the CAPC initiative achieves this transformation by functioning as a credible “Costly Signal” (22) rather than mere rhetoric. Unlike general environmental guidelines, the *CAPC* policy mandates substantial fiscal investments in adaptive infrastructure, such as urban drainage networks and emergency shelters. These tangible physical interventions validate the severity of local climate threats, bridging the psychological distance for citizens. Recent empirical evidence from Luo et al. (23) confirms that this policy constitutes a regulatory shock strong enough to alter firms' cash holding strategies, indicating that market actors decode these investments as credible commitments to climate governance.

Once this credibility is established, pilot cities function as social amplification stations (24) to diffuse awareness. The policy explicitly mandates a management system with “multi-subject participation,” institutionalizing risk communication through community drills and public warning platforms. This mechanism initiates a “State-to-Society Vertical Diffusion” of environmental norms, where campaign style mobilization effectively transforms technical risk data into broad based social consensus. As verified by recent studies, such “model piloting” significantly fosters environmental awareness compared to non-pilot regions. Consequently, the awakened public exerts informal regulatory pressure through green consumption and social supervision, compelling enterprises to adopt cleaner technologies to maintain their legitimacy.

**Hypothesis 2 (H2):** *The CAPC policy promotes urban pollution reduction and carbon emission mitigation by enhancing Public Climate Risk Perception through the mechanisms of costly policy signaling and social amplification of risk*.

#### 2.2.3 The porter hypothesis channel: Green Technology Innovation

Drawing on the Porter Hypothesis, we argue that the *CAPC* policy stimulates Green Technology Innovation (*GTI*) through an “Innovation Compensation Effect” (25).

Compliance Cost Mechanism: The policy instills rigid climate adaptive guidelines in the market entry and license to produce. These raised standards make it costly to comply with high-pollution companies. To keep alive, a rational company must find a way to innovate its production processes through measures such as energy substitution and circular economy technology in order to cover these costs (26–28).

Financing Incentive Mechanism: Pilot policy is usually supported with any of the aforementioned supportive financial means, like green credit and R&D subsidies. These subsidies ease financing pressures on green R&D that is undertaken with high risks and reduce barriers to innovation (29). The subsequent advances in technology do not only make the sources of power more efficient in their use, but also result in a more efficient end of the pipe treatment system, which offers a systemic solution to reducing emissions. Taken together, these mechanisms of innovation compensation lead to the proposal of Hypothesis 3.

**Hypothesis 3 (H3):** *The CAPC policy promotes urban pollution reduction and carbon emission mitigation by stimulating Green Technology Innovation, consistent with the Porter Hypothesis*.

**Figure 1:**
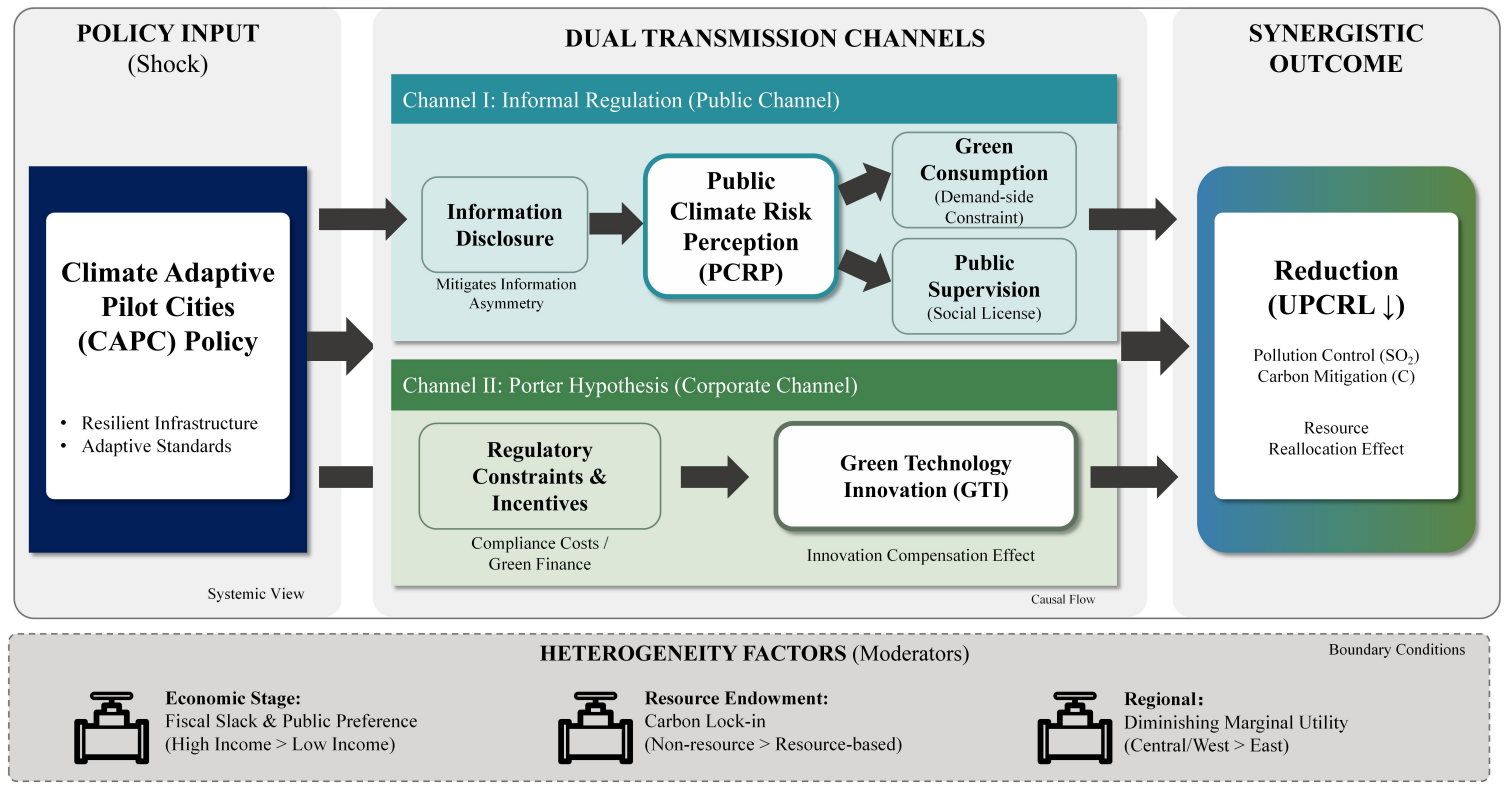
Framework: integrated impacts of climate adaptation on pollution and carbon reduction.

Figure 1 illustrates the conceptual framework of these mechanisms.

## 3 Research design

### 3.1 Baseline model

A quasi-natural experiment of determining the effects of adaptation strategies on urban pollution reduction and carbon emission mitigation is provided by the Climate Adaptive Pilot Cities (CAPC) policy generated (10). To some degree, the biases of self-selection are alleviated through the selection mechanism of pilot cities in which case the provincial recommendation and central review are conducted. To enhance the precision of our causal inference regarding how the CAPC mandate impacts the urban pollution and carbon reduction synergy, we implement a difference-in-differences (DID) framework. The baseline econometric specification is formulated as follows:


$$\begin{array}{l} UPCRL_{it}\;={\;\alpha }_{0}\;+{\;\alpha }_{1}CAPC_{it}\;+\;\alpha_{2}X_{it\;}+\;\mu_{i\;}+\;\nu_{t\;}+\;\varepsilon_{it} \end{array}$$ (1)

In this setup, the indices *i* and *t* denote individual municipalities and specific years, respectively. The regressand, *UPCRL*_*it*_ it characterizes the combined constraint on pollution control and carbon mitigation. The core independent variable, *CAPC*_*it*_, is a dummy variable defined as *Treat*_*i*_ × *Post*_*t*_. *Treat*_*i*_ equals 1 if city *i* is a designated pilot city, and 0 otherwise. *Post*_*t*_ is a time dummy that equals 1 for the year 2017 and subsequent years, and 0 before 2017. *X*_*it*_ represents a vector of control variables. *u*_*i*_ and ν_*t*_ denote city fixed effects and year fixed effects, respectively, to control for time-invariant city characteristics and macroeconomic shocks. ε_*it*_ is the random error term. The coefficient α_1_ captures the net effect of the *CAPC* policy; a significantly negative α_1_ would indicate that the policy effectively promotes urban pollution reduction and carbon emission mitigation.

### 3.2 Variable definitions

#### 3.2.1 Dependent variable urban pollution reduction and carbon emission mitigation (UPCRL)

We construct an urban pollution reduction and carbon emission mitigation (*UPCRL*) index to measure the overall emission levels of carbon and pollutants. Given that China's energy consumption structure is dominated by coal, sulfur dioxide (*SO*_2_) is selected as the representative atmospheric pollutant. Carbon emissions are calculated following the methodology proposed by Wu et al. (30). Distinct from traditional population-based measures, we normalize both *SO*_2_ and carbon emissions scaled by real GDP per capita to ensure comparability across different urban scales. Subsequently, the *UPCRL* index is derived by taking the natural log of the interaction between these two intensity-based metrics. Given the physical nature of *SO*_2_and *CO*_2_ as “joint outputs” of energy consumption, adopting the logarithmic form of the interaction term enables a more accurate capture of the generalized constraint effects of the policy on the urban environmental system. A lower *UPCRL* value indicates better performance in urban pollution reduction and carbon emission mitigation.

#### 3.2.2 Independent variable CAPC policy (CAPC)

This is the interaction term of the pilot city dummy and the time of policy implementation dummy as stated in the equation below; It estimates the mean change in treatment (the climate adaptive policy) on the treated cities compared to the control group.

#### 3.2.3 Mechanism variable

To further elucidate the internal causal pathways through which the policy operates, we identify two primary mechanistic factors: Public Climate Risk Perception (*PCRP*) and Green Technological Innovation (*GTI*).

Public Climate Risk Perception (*PCRP*): In order to estimate this variable, we use the index calculated by Sun et al. (31) as the expression of the Public Views on Climate Risk. This data comes out of the open message board on Leaders (MBL) which is a well-known national online political engagement site (32). This high-frequency index is made up of Natural Language Processing (NLP) techniques constructed by Xu et al. (33). In particular, the authors used the word embedding models in order to construct climate-specific feature and loss-concern dictionaries. They measured the level of public concern by text segmentation and sentiment analysis of the messages of citizens. We also use the aggregation of this index by city-year where a bigger value means the higher the level of awareness and concern of the people about climate risks (34).

Green Technology Innovation (*GTI*): Urban-level green innovative capability is proxied by the volume of green invention patent filings adjusted for population size. Patent applications are preferred over grants as they reflect the firm's immediate innovation response to policy shocks without the time lag associated with the approval process.

#### 3.2.4 Control variables

Following the established empirical frameworks for climate-resilient city evaluations (11, 35), we control for a set of city-level characteristics to mitigate omitted variable bias:

Economic Development (*LnGDP*): The natural logarithm of per capita GDP.

Financial Development (*FinDev*): The ratio of year-end loan and deposit balances to GDP.

Government Intervention (*GovSize*): The ratio of general public budget expenditure to GDP.

Education Level (*Edu*): The share of education expenditure in total fiscal expenditure.

Consumption Level (*Consum*): The ratio of total retail sales of consumer goods to GDP.

Technological Support (*TechSup*): The share of science and technology expenditure in GDP.

Industrial Structure (*IndStruc*): The ratio of the value-added of the secondary industry to GDP.

### 3.3 Data sources

The empirical foundation of this study is a balanced panel dataset covering 273 Chinese prefecture-level administrative units spanning the 13-year period from 2011 to 2023. Primary socio-economic data are derived from three key publications: *the China City Statistical Yearbook, China Energy Statistical Yearbook, and China Urban Construction Statistical Yearbook*. Patent data are obtained from the *China National Intellectual Property Administration (CNIPA)*. The unique *PCRP* data are derived from the textual analysis of the “Message Board for Leaders” platform (36). To ensure data quality, we apply linear interpolation to fill missing values for a small number of observations. The statistical properties of the primary variables utilized in this analysis are summarized in Table 1.

**Table 1:** Descriptive statistics of variables.

Variable	Symbol	Obs	Mean	Std. dev.	Min	Max
Dependent variable						
*Urban pollution reduction and carbon emission mitigation*	*UPCRL*	3,549	4.0589	1.6892	0.0000	10.1817
Independent variable						
*CAPC* policy	*CAPC*	3,549	0.0454	0.2081	0.0000	1.0000
Control variables						
Economic development	*LnGDP*	3,549	10.8209	0.6981	8.7297	13.1851
Financial development	*FinDev*	3,549	2.6544	1.2714	0.5879	21.3015
Government intervention	*GovSize*	3,549	0.2002	0.0993	0.0439	0.9155
Education level	*Edu*	3,549	0.1745	0.0380	0.0146	0.3562
Consumption level	*Consum*	3,549	0.3807	0.1081	0.0544	1.0126
Technological support	*TechSup*	3,549	0.0031	0.0029	0.0001	0.0688
Industrial structure	*IndStruc*	3,549	0.4335	0.1086	0.1159	0.8934

## 4 Empirical findings

### 4.1 Benchmark regression results

Table 2 presents the baseline regression results for the impact of the CAPC policy on the urban pollution reduction and carbon emission mitigation (*UPCRL*). Columns (1) through (5) show the results with the gradual inclusion of control variables. Column (6) displays the estimation with the full set of city-level controls, as well as city and year fixed effects.

**Table 2:** Baseline regression results.

Variables	*UPCRL*					
	(1)	(2)	(3)	(4)	(5)	(6)
*CAPC*	−0.632^***^ (0.143)	−0.373^***^ (0.142)	−1.888^***^ (0.099)	−0.376^***^ (0.085)	−0.290^***^ (0.079)	−0.284^***^ (0.078)
*LnGDP*		−0.953^***^ (0.070)		−2.500^***^ (0.087)		−0.250^**^ (0.123)
*FinDev*		0.257^***^ (0.038)		−0.324^***^ (0.095)		−0.041 (0.036)
*GovSize*		−4.079^***^ (0.429)		−1.258^*^ (0.748)		1.191^**^ (0.607)
*Edu*		1.635^**^ (0.802)		−0.098 (0.895)		0.989 (0.815)
*Consum*		0.505^*^ (0.277)		1.994^***^ (0.242)		0.848^***^ (0.186)
*TechSup*		−42.685^***^ (9.954)		−2.872 (6.283)		−13.386^**^ (5.67)
*IndStruc*		3.744^***^ (0.354)		4.207^***^ (0.587)		−0.333 (0.353)
Constant	4.088^***^ (0.029)	12.553^***^ (0.867)	4.145^***^ (0.020)	29.681^***^ (0.968)	4.072^***^ (0.011)	6.339^***^ (1.469)
City FE	No	No	Yes	Yes	Yes	Yes
Year FE	No	No	No	No	Yes	Yes
Observations	3,549	3,549	3,549	3,549	3,549	3,549
*R^2^*	0.006	0.154	0.560	0.826	0.862	0.865

Standard errors in parentheses. ^***^, ^**^, and ^*^ denote significance at the 1%, 5%, and 10% levels, respectively.

The results consistently show that the coefficient of the core explanatory variable, *CAPC*, is significantly negative at the 1% level across all specifications. Specifically, in the preferred specification (Column 6), the coefficient is −0.284. Notably, as *UPCRL* is defined as the natural logarithm of the product of *SO*_2_ and *CO*_2_ intensities, the coefficient of −0.284 captures the additive sum of the policy's marginal impacts on both dimensions. Economically interpreted, this coefficient suggests that, holding all other factors constant, the activation of pilot status corresponds to a roughly 0.284 decline in the combined emission intensity for the treated municipalities compared to their non-treated counterparts. This finding empirically supports Hypothesis 1, suggesting that the CAPC policy can effectively achieve the goal of urban pollution reduction and carbon emission mitigation.

### 4.2 Parallel trend test

The validity of the DID framework for causal identification hinges on the common trend prerequisite. This assumption necessitates that, in the counterfactual scenario absent the policy intervention, the temporal evolution of the outcome variable would have followed a symmetrical trajectory for both the experimental and comparison cohorts. Following the event study framework, we estimate the following dynamic model to test this assumption and examine the dynamic effects of the policy:


$$\begin{array}{c} UPCRL_{t}\;={\;\alpha }_{0}\;+\;\overset{6}{\underset{k=-5}{\sum }}\beta_{k}D_{t}^{k}\;+\;\gamma X_{it}\;+{\;\mu }_{i}\;+\;v_{t\;}+\;\epsilon_{it} \end{array}$$ (2)

where $D_{it}^{k}$ represents a set of dummy variables indicating the k-th year relative to the policy launch year (2017). The sample period spans from 2011 to 2023, corresponding to relative time periods from *k* = −6 to = 6. To avoid multicollinearity, we exclude the initial year of the sample (2011, i.e., *k* = −6) as the base period. The coefficient β_*k*_ captures the difference in *UPCRL* between pilot and non-pilot cities in year k relative to the base year.

**Figure 2:**
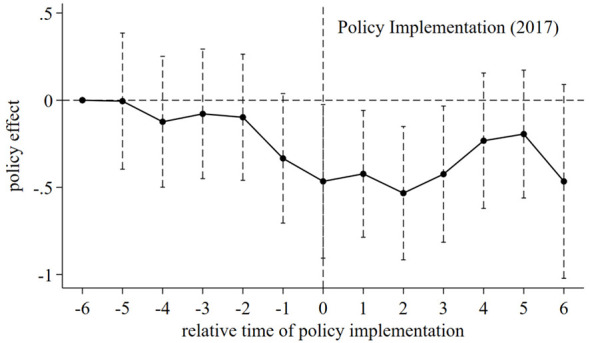
Parallel trend test and dynamic effects of the CAPC policy.

Figure 2 plots the estimated coefficients (β_*k*_) and their 95% confidence intervals.

The results demonstrate that the estimated coefficients for the years prior to the policy implementation (*k* < 0) are statistically insignificant and fluctuate around zero. This confirms that, relative to the base year of 2011, there were no systematic pre-existing divergences in trends between the treatment and control groups, thereby satisfying the parallel trend assumption. In the post-implementation period (*k*≥0), the coefficients become significantly negative and the magnitude of the reduction generally increases over time, indicating that the CAPC policy exerts a persistent and strengthening inhibitory effect on urban pollution and carbon emissions.

### 4.3 Robustness checks

To ensure the reliability of our baseline findings, we conduct a series of robustness checks.

#### 4.3.1 Placebo test

**Figure 3:**
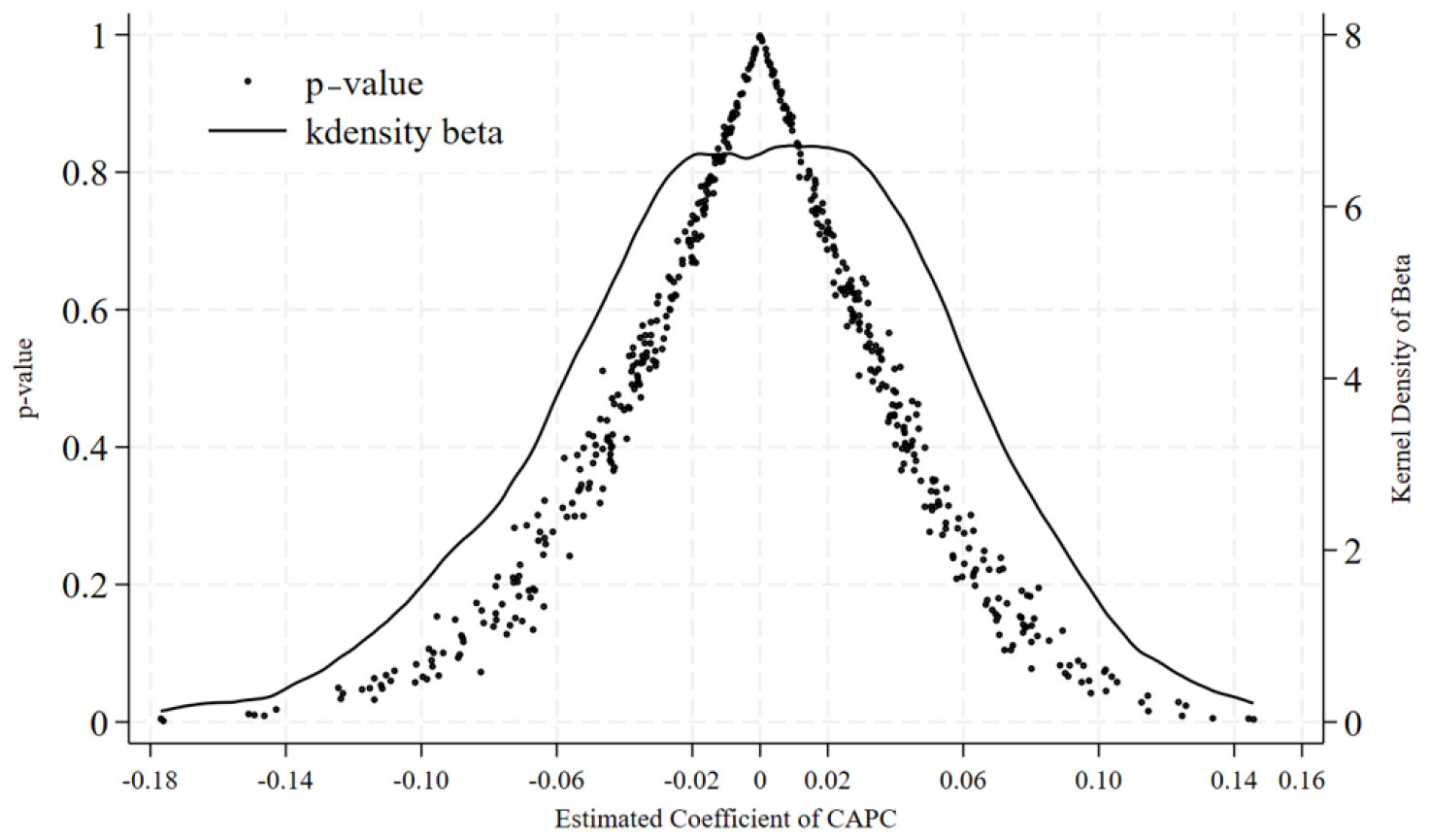
Distribution of estimated coefficients from the placebo test.

In order to dismiss the hypothesis that the observed outcomes are motivated by invisible random variables, we obtain a placebo test by assigning treatments randomly. We randomly designate the pilot status of a group of cities and come up with a pseudo treatment time, and repeat it 500 times. The kernel density distribution of the estimated coefficients values of the pseudo-interaction terms is shown in Figure 3. The distribution is very similar to a normal distribution with a mean at zero and the coefficient obtained at the baseline regression (−0.284) is far on the left tail which substantially deviates the placebo distribution. This fact indicates that random chance and unobserved confounders are not the chief factors that are behind our key findings.

#### 4.3.2 PSM-DID approach

**Figure 4:**
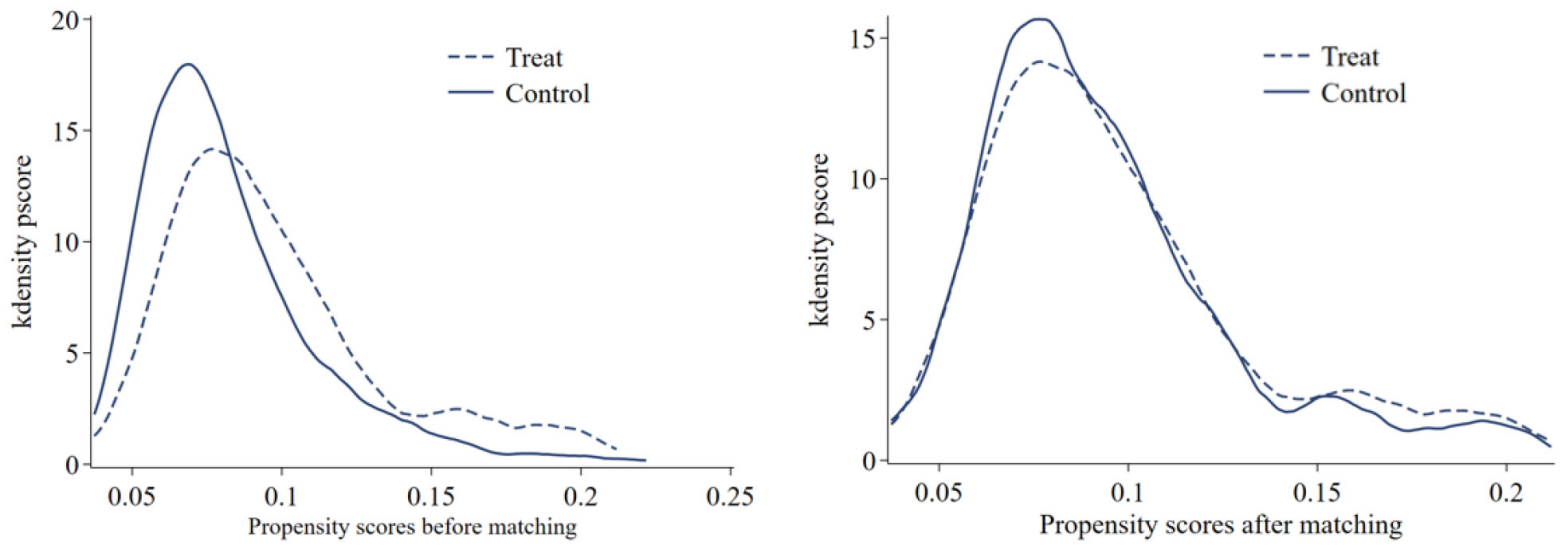
Distribution of propensity scores before and after matching.

To mitigate potential self- potential endogeneity issues stemming from the non-stochastic allocation of pilot cities (e.g., cities with better infrastructure might be more likely to be selected), we employ the Propensity Score Matching combined with Difference-in-Differences (PSM-DID) method. We use the control variables as covariates and apply the 1:3 nearest neighbor matching algorithm with a caliper to match pilot cities with comparable non-pilot cities. After matching, the covariate distribution exhibits a high degree of balance between the treated and untreated groups (see Figure 4). Subsequently, we perform the DID estimation on this propensity-matched dataset. As detailed in Table 3, the estimator for the *CAPC* variable retains its statistical significance (at the 10% level) and negative sign, confirming that our findings are robust to potential selection bias.

**Table 3:** PSM-DID regression results.

Variables	*UPCRL*			
	(1)	(2)	(3)	(4)
*CAPC*	−0.632^***^ (0.143)	−0.361^**^ (0.140)	−0.407^***^ (0.084)	−0.294^***^ (0.079)
Constant	4.104^***^ (0.029)	13.447^***^ (0.906)	27.939^***^ (0.947)	7.194^***^ (1.642)
Controls	No	Yes	Yes	Yes
City FE	No	No	Yes	Yes
Year FE	No	No	No	Yes
Observations	3,425	3,425	3,425	3,425
*R* ^2^	0.006	0.170	0.840	0.868

Standard errors in parentheses. ^***^, ^**^, and ^*^ denote significance at the 1%, 5%, and 10% levels, respectively. The sample is matched using PSM.

#### 4.3.3 Median regression

To ensure the conclusions are not driven by specific sample distributions, we re-estimate the model using Median Regression, which provides a more resistant characterization of the policy's impact (37). As shown in Column (1) of Table 4, the results remain significantly negative and highly consistent with the baseline, confirming the distributional robustness of our findings.

**Table 4:** Robustness checks.

Variables	***UPCRL***					
	Alternative specifications			Net effect analysis		
	(1)	(2)	(3)	(4)	(5)	(6)
**Model specification**	**Median regression**	**Omitting anticipation**	**Lagged term**	**Control for sponge city**	**Control for smart city**	**Control for low-carbon city**
*L.CAPC*			**–**0.195^**^ (0.083)			
*CAPC*	−0.260^***^ (0.121)	−0.334^***^ (0.089)		−0.251^***^ (0.079)	−0.286^***^ (0.078)	−0.291^***^ (0.078)
Constant	1.617 (1.507)	6.403^***^ (1.598)	6.467^***^ (1.597)	6.385^***^ (1.464)	6.281^***^ (1.464)	6.408^***^ (1.470)
Controls	Yes	Yes	Yes	Yes	Yes	Yes
City FE	Yes	Yes	Yes	Yes	Yes	Yes
Year FE	Yes	Yes	Yes	Yes	Yes	Yes
Observations	3,549	3,003	3,476	3,549	3,549	3,549
*R* ^2^	0.681	0.864	0.864	0.865	0.865	0.865

Standard errors in parentheses. ^***^, ^**^, and ^*^ denote significance at the 1%, 5%, and 10% levels, respectively.

#### 4.3.4 Excluding the anticipated effect

To alleviate potential interference from pre-treatment policy signals (i.e., candidate cities might proactively adjust prior to formal approval), we omit the samples from the 2 years before policy implementation to exclude the anticipated effect. As evidenced by the regression outputs in Column (2) of Table 4, the coefficient for the core policy variable (*CAPC*) persists in being significantly negative. This robustness check further corroborates that the integrated emission-reduction effect is driven by the actual policy shock rather than pre-existing anticipated trends.

#### 4.3.5 Lagged explanatory variable

To alleviate potential endogeneity concerns caused by reverse causality (i.e., cities with lower emissions might be selected as pilots), we lag the core independent variable by one period (*L*.*CAPC*). As evidenced by the regression outputs in Column (3) of Table 4, the coefficient for the one-period lagged policy variable (*L*.*CAPC*) persists in being significantly negative. This robustness check further corroborates the causal orientation of our baseline conclusions.

#### 4.3.6 Excluding confounding policies (net effect analysis)

During the sample period, several other environmental pilot policies were implemented in China, which might confound our estimates. To isolate the net effect of the CAPC policy, we explicitly control for three overlapping policies:

Low-Carbon City Pilot Policy: Directly targets carbon emission reduction.

Smart City Pilot Policy: May affect emissions through technological optimization (38).

Sponge City Pilot Policy: Closely related to climate adaptation infrastructure.

We introduce dummy variables for these policies into the baseline model. The results in Columns (4)–(6) of Table 4 demonstrate that, even after controlling for these confounding policies, the coefficient of the CAPC policy remains significantly negative and stable. This strongly suggests that the overall reduction effect is driven by the CAPC policy itself rather than by other concurrent environmental initiatives.

## 5 Mechanism analysis

With that determination, that since the CAPC policy does much to foster the urban pollution reduction and carbon emission mitigation, we enter the black box of its transmission process. Following the theoretical model, our hypothesis is that the policy achieves its effect on two main levels Informal Regulation (based on the perception of public the climate risk) and Technological. Innovation (based on the Hypothesis of Porter). Using the step approach of causation we estimate the following models to test these mediating effects:


$$\begin{array}{l} Mediator_{it}\;=\beta_{0}\;+{\;\beta }_{1}CAPC_{it}\;+\;\beta_{2}X_{it}\;+\;\mu_{i}\\ \;++\;\nu_{t}\;+{\;\epsilon }_{it} \end{array}$$ (3)

$$\begin{array}{c} UPCRL_{it}\;=\gamma_{0}\;+\;\gamma_{1}CAPC_{it}\;+{\;\gamma }_{2}Mediator_{it}\\ +{\;\gamma }_{3}X_{it}\;+{\;\mu }_{i}\;+{\;\nu }_{t}\;+{\;\epsilon }_{it} \end{array}$$ (4)

where *Mediator*_*it*_ represents either Public Climate Risk Perception (*PCRP*) or Green Technology Innovation (*GTI*).

### 5.1 The informal regulation channel: Public Climate Risk Perception

We initially investigate on whether the policy of CAPC can add value to the environmental governance through increase in awareness among the populace, which serves as an informal regulation mechanism. The findings of the regression are provided in Columns (2) and (3), Table 5.

**Table 5:** Mechanism analysis results.

Variables	(1)	(2)	(3)	(4)	(5)
	*UPCRL*	*PCRP*	*UPCRL*	*GTI*	*UPCRL*
*CAPC*	−0.284^***^ (0.078)	0.487^***^ (0.183)	−0.259^***^ (0.077)	0.135^***^ (0.043)	−0.246^***^ (0.078)
*PCRP*			−0.052^***^ (0.008)		
*GTI*					−0.282^***^ (0.026)
*LnGDP*	−0.250^**^ (0.123)	−1.005^**^ (0.418)	−0.302^**^ (0.122)	0.039 (0.072)	−0.239^**^ (0.121)
*FinDev*	−0.041 (0.036)	0.164^***^ (0.056)	−0.033 (0.036)	0.032^***^ (0.010)	−0.032 (0.036)
*GovSize*	1.191^**^ (0.607)	−3.883^***^ (1.404)	0.988^*^ (0.600)	−0.634^**^ (0.272)	1.012^*^ (0.590)
*Edu*	0.989 (0.815)	2.080 (1.304)	1.097 (0.813)	3.208^***^ (0.477)	1.894^**^ (0.819)
*Consum*	0.848^***^ (0.186)	−2.396^***^ (0.407)	0.723^***^ (0.187)	−0.872^***^ (0.111)	0.602^***^ (0.187)
*TechSup*	−13.386^**^ (5.674)	−8.931 (15.310)	−13.851^**^ (5.689)	37.309^***^ (14.109)	−2.858 (5.656)
*IndStruc*	−0.333 (0.353)	1.204^*^ (0.690)	−0.271 (0.350)	0.255 (0.172)	−0.262 (0.350)
Constant	6.339^***^ (1.469)	11.924^**^ (4.751)	6.959^***^ (1.460)	−0.375 (0.787)	6.233^***^ (1.449)
Indirect effect (Sobel)			−0.025^*^ (0.010)		−0.038^**^ (0.013)
City FE	Yes	Yes	Yes	Yes	Yes
Year FE	Yes	Yes	Yes	Yes	Yes
Observations	3,549	3,549	3,549	3,549	3,549
*R^2^*	0.865	0.460	0.867	0.818	0.869

Standard errors in parentheses. ^***^, ^**^, and ^*^ denote significance at the 1%, 5%, and 10% levels, respectively.

Under Column (2), where the dependent variable is *PCRP*, the coefficient of *CAPC* = 0.487 and has a significant value at 1 percent level. This means that the implementation of the policy via machine like climate risks warning, and information disclosure have been able to create awareness among people about environmental risks (39, 40).

Column (3) is where we incorporate *PCRP* in the *UPCRL* model of understanding. *PCRP* coefficient is also considerably negative (−0.052) indicating that an increased public risk perception corresponds to successful pressure to achieve an emission reduction (e.g., green consumption decision or social check) (36, 41, 42). It is interesting to note that the coefficient of *CAPC* remains negative with significant negative value (−0.259), but the value dropped in absolute value relative to the baseline coefficient (−0.284). This means that Public Climate Risk Perception is a partial mediator. Sobel test once again motivates the importance of this mediation path (0.1 % significant Z-statistic) which proves Hypothesis 2.

### 5.2 The porter hypothesis channel: Green Technology Innovation

Next, we test the “Porter Hypothesis,” investigating whether the policy triggers an “innovation compensation effect.” The results are presented in Columns (4) and (5) of Table 5.

As evidenced in Column (4), the regression results reveal that the *CAPC* initiative serves as a robust catalyst for Green Technology Innovation (*GTI*). The estimated parameter is 0.135 (*p* < 0.01), confirming a statistically significant promotional effect (43). This suggests that the regulatory constraints and fiscal incentives associated with the pilot policy have induced firms to increase R&D investment in green technologies.

In Column (5), the coefficient of *GTI* on *UPCRL* is significantly negative (−0.282), indicating that technological progress is a potent driver of the urban pollution reduction and carbon emission mitigation. Similar to the previous channel, the coefficient of *CAPC* decreases in magnitude from −0.284 (Baseline) to −0.246 (Column 5), confirming that Green Technology Innovation partially mediates the relationship between the CAPC policy and low-carbon development (44). The significant result of the Sobel test supports this causal chain, validating Hypothesis 3.

## 6 Heterogeneity analysis

The local endowments of resources as well as economic stages of development are closely interconnected with the efficiency of the CAPC policy to reduce the urban pollution reduction and carbon emission mitigation. As there are significant differences among the cities in China, to reveal the boundary framework of the policy effectiveness, we make heterogeneity analyses based on the geographic location, economic development level and resource dependence.

### 6.1 Regional heterogeneity

We separate the sample into Eastern region, Central region and Western region. Regression outcomes in Table 6 indicate that there are both large and small deviations in the coefficients of the *CAPC* which are significantly negative in the Central (−0.560, *p* < 0.01) and Western (−0.343, *p* < 0.05) regions, but have statistically insignificant coefficients in the Eastern region.

**Table 6:** Regional heterogeneity analysis.

Variables	Eastern	Central	Western
*CAPC*	−0.036 (0.102)	−0.560^***^ (0.083)	−0.343^**^ (0.157)
*LnGDP*	−0.108 (0.210)	0.179 (0.184)	−0.985^***^ (0.246)
*FinDev*	−0.132^**^ (0.053)	−0.060 (0.053)	0.094^**^ (0.036)
*GovSize*	3.694^***^ (1.149)	2.810^***^ (0.827)	−1.599^*^ (0.935)
*Edu*	0.606 (1.145)	0.271 (1.336)	0.799 (1.786)
*Consum*	1.032^***^ (0.307)	1.096^***^ (0.278)	0.132 (0.454)
*TechSup*	−13.693 (9.352)	−5.067 (17.997)	−13.139^*^ (7.642)
*IndStruc*	−0.120 (0.802)	−1.159^**^ (0.518)	−0.245 (0.595)
Constant	4.860^*^ (2.586)	1.610 (2.097)	14.608^***^ (2.880)
City FE	Yes	Yes	Yes
Year FE	Yes	Yes	Yes
Observations	1,261	1,261	1,027
*R^2^*	0.898	0.871	0.829

Standard errors in parentheses. ^***^, ^**^, and ^*^ denote significance at the 1%, 5%, and 10% levels, respectively.

The law of diminishing marginal utility in pollution abatement provides a primary explanation for this spatial divergence. Eastern cities, having undergone extensive industrial upgrading and strict environmental regulation, have largely exhausted the “low-hanging fruits” of emission reduction. This results in a state of “policy saturation,” where higher marginal abatement costs prevent additional gains from soft pilot initiatives. In contrast, the Central and Western regions, often located in ecologically vulnerable areas with relatively weaker foundations for green infrastructure, enjoy distinct potential for the CAPC policy to drive measurable reductions (45). These regions exhibit higher sensitivity to regulatory stimuli due to their vast untapped marginal abatement space. Notably, the significance in these areas is fundamentally driven by regional hub cities that possess both extensive abatement potential and the robust policy execution capacity required to implement adaptive mandates.

### 6.2 Economic development heterogeneity

Using per capita GDP tertiles, we categorize cities into Low, Medium, and High economic levels. According to the empirical estimates presented in Table 7, the pilot program delivers a statistically significant abatement effect exclusively within the high-income cohort, where the coefficient reaches −0.395 (significant at the 1% level). Conversely, the policy's influence remains statistically negligible across the low- and medium-income sub-samples.

**Table 7:** Economic development heterogeneity analysis.

Variables	Low economic level	Medium economic level	High economic level
*CAPC*	−0.250 (0.192)	−0.025 (0.102)	−0.395^***^ (0.100)
*LnGDP*	0.185 (0.205)	−1.196^***^ (0.259)	−0.035 (0.202)
*FinDev*	−0.015 (0.025)	−0.085 (0.099)	−0.164^**^ (0.080)
*GovSize*	0.825 (0.899)	−0.218 (0.929)	3.157^**^ (1.525)
*Edu*	0.626 (1.542)	2.972^**^ (1.398)	1.872 (1.382)
*Consum*	0.614^**^ (0.258)	−0.266 (0.413)	2.346^***^ (0.360)
*TechSup*	−17.778^**^ (7.765)	13.272 (18.411)	3.163 (14.991)
*IndStruc*	−1.378^**^ (0.667)	0.521 (0.568)	0.723 (0.685)
Constant	2.270 (2.267)	16.578^***^ (3.067)	2.849 (2.637)
City FE	Yes	Yes	Yes
Year FE	Yes	Yes	Yes
Observations	1,183	1,183	1,183
*R* ^2^	0.820	0.878	0.903

Standard errors in parentheses. ^***^, ^**^, and ^*^ denote significance at the 1%, 5%, and 10% levels, respectively.

We attribute these findings to a “Capacity Threshold” mechanism. High-income cities possess superior endowments of human capital, R&D infrastructure, and “fiscal slack,” providing the necessary capacity to react to innovation incentives and fund capital-intensive infrastructure (46). Consistent with the Environmental Kuznets Curve (EKC) hypothesis and Maslow's hierarchy, residents in affluent cities exhibit a stronger preference for environmental quality, thereby amplifying informal regulatory pressure through public risk perception. Conversely, low-income cities are often constrained by survival-first priorities and limited innovation capacity, making it difficult to effectively mobilize reduction channels (47). These results establish a “Capacity-Space” framework: although high-income cities are intuitively associated with the East, our sample reveals a critical “geo-economic intersection” where many provincial capitals and regional hub cities in the Central and Western regions possess high-income characteristics. Policy efficacy is maximized only when a city simultaneously crosses the “Capacity Threshold” and possesses the “Space Threshold” (marginal potential), illustrating how the interaction between economic capacity and geographic potential drives aggregate performance.

### 6.3 Resource dependence heterogeneity

Last, Table 8 indicates that only the CAPC policy could lead to a large decrease in emissions in Non-resource-based cities only (−0.378, *p* < 0.01).

**Table 8:** Resource dependence heterogeneity analysis.

Variables	Resource-based cities	Non-resource-based cities
*CAPC*	−0.102 (0.189)	−0.378^***^ (0.073)
*LnGDP*	−0.413^**^ (0.201)	−0.202 (0.186)
*FinDev*	−0.175 (0.112)	−0.029 (0.026)
*GovSize*	0.022 (0.815)	3.480^***^ (0.847)
*Edu*	0.729 (1.348)	1.590^*^ (0.954)
*Consum*	0.286 (0.281)	1.471^***^ (0.253)
*TechSup*	−6.611 (4.475)	−24.275^*^ (12.546)
*IndStruc*	−0.886^*^ (0.497)	0.285 (0.508)
Constant	9.183^***^ (2.411)	4.737^**^ (2.150)
City FE	Yes	Yes
Year FE	Yes	Yes
Observations	1,443	2,106
*R* ^2^	0.841	0.885

Standard errors in parentheses. ^***^, ^**^, and ^*^ denote significance at the 1%, 5%, and 10% levels, respectively.

This result supports the existence of a “Carbon Lock-in Effect” (48). The feature of resource-based cities is that a strict form of the industrial organization heavily relying on the extraction of fossil fuels and their processing (49). High barriers to transformation are formed by this dependence on paths. The CAPC policy soft constraints do not always have the necessary power to breach the hard emissions of the leading heavy industries (50). On the other hand, the non-resource-based cities, which have more adaptable industrial frameworks, can better utilize the pilot policy to promote green innovation and resource allocation optimization, thereby achieving urban pollution reduction and carbon emission mitigation.

## 7 Conclusions and policy implications

This paper views the creation of Climate Adaptive Pilot Cities (CAPC) as a quasi- natural experiment in order to empirically study the potential promotional effects of climate adaptation on urban pollution reduction and carbon emission mitigation. In the form of this work, employing a dynamic staggered difference-in-differences model, based on a panel dataset of 273 Chinese cities in 2011 to 2023, we are able to conclude that the CAPC policy indeed leads to a great improvement in the level of urban pollution reduction and carbon emission mitigation, which lowered cities' pollution and carbon emission index by 28.4 percent. This composite abatement effect is observed even after the removal of co-existing environmental policy justifying that climate resilience building does not necessarily imply a trade-off with climate mitigation objectives. In our mechanism analysis, we will identify two channels of transmission, including the “informal regulation” channel, where the policy increases the perceived risk of climatic conditions in the population, which has just been confirmed with novel text-mining data, to create bottom-up supervision pressure; and the “Porter Hypothesis” channel, where regulation restrictions lead to green technology generation, thus improving energy efficiency at the source.

The effectiveness of the policy is, however, not as comparable in all contexts. It is at the stage of local resource endowment and economic level that we find considerable heterogeneity. The law of diminishing marginal effect of pollution abatement has a greater marginal effect in the Central and Western urban areas by the policy. On the other hand, in high-income urban areas, where it works well, the importance of so-called fiscal capacity can be seen; such urban areas have financial slack to afford expensive adaptation infrastructure. It is interesting to note that the outcome of resource-based cities is insignificant which confirms the hypothesis of carbon lock-in, when strict industrial framework dilutes the impact of weak pilot policies.

On the basis of these findings, policymakers would be advised to emphasize the orientation of the city-planning standards to log along with the incorporation of the so-called low-carbon adaptation standards to avoid maladjustment. Carbon assessment indicators must be included in the acceptability of resilient infrastructure so that mitigation measures are also met by adaptation measures. Moreover, since the perception of the population is a critical factor, local governments ought to use digital platforms to make climate risk communication institutional. The authorities can address the cognitive gap by observing the social opinion and mobilizing the role of the population in the informal control. Last but not least, the heterogeneity problem encountered should be addressed with differentiated support mechanisms. For fiscally with constrained regions, more specific climate adaptation fund needs to be implemented by the central government to deal with the issue of the un-funded mandate, whereas more robust hard constraints and just transition support is material in the cities that are based on resources to break past the structural barriers.

There are weaknesses in this research. To begin with, we analyze the data at the city scale because of the availability of data; further studies may use firm-level data to study the micro-level options of adaptation to change. Second, although we put the emphasis on local treatment impacts, future research can consider the power of spatial econometric models over the possibility of cross-border environmental spillovers of adaptation policies.

## Data Availability

The original contributions presented in the study are included in the article/supplementary material, further inquiries can be directed to the corresponding authors.
